# Expansion of the application domain of a macromolecular ocular irritation test (OptiSafe^™^)

**DOI:** 10.1016/j.tiv.2022.105515

**Published:** 2022-11-06

**Authors:** Stewart Lebrun, Sara Chavez, Linda Nguyen, Roxanne Chan

**Affiliations:** Lebrun Labs LLC, Anaheim, CA, United States of America

**Keywords:** Ocular irritation, Validation, Application domain, OptiSafe, Nonanimal eye irritation test

## Abstract

The OptiSafe (OS) test is shelf-stable, macromolecular eye irritation test that does not include any animal ingredient or component (“vegan”). The purpose of this study was to evaluate the test’s accuracy for an expanded application domain for both the original and recently updated OS method. This study involved the testing of additional ocular corrosives and previously excluded foaming agents (“surfactants”) using both the original and updated OS methods and then combining these data with prior validation data for a total of 147 chemicals. Predictivity was evaluated by a statistical comparison of the OptiSafe predictions with historical in vivo “Draize” rabbit eye data for the same chemicals (from public databases). We report that for the detection of chemicals not requiring classification for eye irritation [Globally Harmonized System of Classification and Labeling of Chemicals (GHS) No Category], the accuracy, specificity, and sensitivity were 92.8%, 79.6%, and 100.0%, respectively, for the updated method; for the detection of chemicals inducing extreme eye damage/corrosion (GHS Category 1), the accuracy, specificity, and sensitivity were 79.4%, 71.8%, and 91.7%, respectively, for the updated method. Results indicate that both the original and updated methods have a high accuracy for the expanded application domain that included ocular corrosives and surfactants.

## 1. Introduction

Chemically induced eye damage has traditionally been evaluated using a live rabbit eye test. This test (“Draize test”) involves instilling 100 μL of the substance under evaluation within the conjunctival sac of a live New Zealand White rabbit (Draize et al., 1944). Indices of toxicity (redness, swelling, opacity of the cornea, discharge, lesions) are clinically graded and recorded for the cornea, iris, and conjunctiva daily for up to 21 days (Luechtefeld et al., 2016; OECD, 2021a). Based on these toxicity outcomes, chemicals can be classified using the Globally Harmonized System of Classification and Labeling of chemicals (GHS) of eye irritation classification (UN, 2021). The GHS classification system includes the category “not classified” (NC; chemicals that do not induce significant levels of eye irritation or damage averaged over the first three days after exposure) (UN, 2021), Categories 2B and 2A “ocular irritants” (chemicals that induce significant irritation over the first three days but then the irritation reverses prior to 7 days (2B) or 21 days (2A), [UN, 2021]), and Category 1 “ocular corrosives” (chemicals that cause an extreme response or corrode and permanently damage the eye) (UN, 2021).

Since the use of live animals for routine product testing is not consistent with efforts to “reduce, refine, and replace” animal studies (Liebsch et al., 2011), there is a strong shift from animal testing toward the use of nonanimal test methods (Humane Society, 2013). A number of nonanimal eye toxicity tests have been recognized by the Organization for Economic Cooperation and Development (OECD) for which test guidelines have been established, including in vitro cell culture-based tests that apply test substances to a cell monolayer and determine if there is a reduction in viability using the 3-(4,5-dimethylthiazol-2-yl)-2,5-diphenyltetrazolium bromide (MTT) assay. [Short Time Exposure (STE) test] and ex vivo tests that use food-source animal eyes, which includes the Isolated Chicken Eye (ICE) and the Bovine Corneal Opacity and Permeability (BCOP) tests (OECD, 2018, 2020a; Verstraelen et al., 2017; Lebrun et al., 2021). For both types of tests, materials are applied to the corneal surface, and damage is assessed by measuring corneal opacity, swelling (ICE), and fluorescein staining (ICE) or fluorescein permeability (BCOP). Additionally, OECD test guidelines exist for in vitro reconstituted human corneal epithelial (rhCE) tests, in which materials are applied to 3D epithelial cultures (EpiOcular^™^, SkinEthic^™^, and MCTT HCE^™^ Eye Tests), and viability is measured using the MTT assay (Pfannenbecker et al., 2013; OECD, 2019a; Kandarova et al., 2018; Van Rompay et al., 2018; Lim et al., 2019). Recently, a new protocol (Time-To-Toxicity; TTT) was developed for the reconstituted corneal epithelium equivalent test SkinEthic. This procedure involves dosing tissues for 6, 16, and 120 min for liquids or 30 and 120 min for solids and using MTT-based viability data to predict the level of irritation (Alépée et al., 2021, 2022). There is also a long list of other alternative tests that have not received OECD acceptance (Bagley et al., 1999; Hafner, 2000; Piehl et al., 2011; Bartok et al., 2015; Spöler et al., 2015; Adriaens et al., 2018; Araujo Lowndes Viera et al., 2022).

Emerging alternatives to animal testing are the “macromolecular” tests. These test tube (“*in chemico*”) methods are highly standardized and shelf-stable options for the assessment of eye irritation potential. Opti-Safe^™^ (“Optimized for Safety”) is a shelf-stable in chemico test method that consists of a proprietary macromolecular test matrix that is used to quantify the potential of an unknown test material to cause eye irritation or eye damage. Damage to macromolecules results in the loss of cell viability, and the extent of measured macromolecular damage is used to predict the toxicity of the material being tested (Lebrun, 2018; 2021; Lebrun et al., 2021a, 2021b; Lebrun et al., 2022; Choksi et al., 2020; Lebrun et al., 2019, 2021a, 2021b, 2022a, 2022b).

To conduct the test, materials to be tested are added to “ocular discs” to control the delivery of the chemical to be tested as it enters the macromolecular reagent mixture (insoluble materials are floated instead of placed within membrane discs). Results are read using a spectro-photometer and the optical density (OD; at 400 nm) and pH values are compared with quality controls and a standard curve to calculate the irritation score. The score is then applied to a prediction model to classify the material tested. For a detailed description, see Lebrun et al., 2022a.

A validation study of the OS test was coordinated by the National Toxicology Program Interagency Center for the Evaluation of Alternative Toxicological Methods, with members of Interagency Coordinating Committee on the Validation of Alternative Methods (ICCVAM) as the validation management team that assessed the test’s accuracy for the detection of nonirritants versus irritants (GHS NC versus the rest). Foaming materials (“surfactants”) were excluded from the study, and there were only 11 GHS Category 1 chemicals. For the three-lab blind transferability phase, when results were combined based on the majority classification for the GHS classification, the accuracy was 89%, the false-negative (FN) rate was 0%, the false-positive (FP) rate was 23%, the sensitivity was 100%, the specificity was 77%, and the balanced accuracy was 88.5% (Choksi et al., 2020). Additional chemicals were selected by ICCVAM for evaluation of the test’s application domain; these materials were tested by the lead lab only in the “application domain phase.” Based on overall results from the three-lab blind transferability study and the blind application domain study, the test method accuracy for the GHS system was 80%, the FN rate was 0%, the FP rate was 40%, the sensitivity was 100%, the specificity was 60%, and the balanced accuracy was 80% (Lebrun et al., 2021a).

We then studied the overpredicted chemicals (Lebrun et al., 2021a) and found that chemicals associated with reactive oxygen species (ROS) chemistry were commonly overpredicted (Lebrun et al., 2021b, 2022a). Based on the hypothesis that naturally occurring antioxidants in tears can deactivate ROS prior to eye damage, we then tested a range of tear antioxidants and identified that ascorbic acid reduces the OptiSafe FP rate (Lebrun et al., 2021b, 2022a) and then compared results for the same chemicals with and without ascorbic acid (Lebrun et al., 2022b). We then updated the prediction model, the OS physiochemical handling procedures (PCHPs) and also comprehensively determined the impact of adding an antioxidant to the OS test matrix (Lebrun et al., 2021b, 2022a). All prior OS-coded validation study chemicals were then retested (Lebrun et al., 2022a). These chemicals included the prior coded validation chemicals described above (Choksi et al., 2020; Lebrun et al., 2021a). Based on the retesting of chemicals, for the detection of GHS NC, the addition of an antioxidant to levels found in tears lowered the FP rate from 40.0% to 22.2%. The FN rate remained the same at 0.0% (100% sensitivity), and the overall accuracy improved from 80.3% to 89.2% (Lebrun et al., 2022a); however, this study only evaluated the accuracy for GHS NC chemicals versus the rest, and no surfactants were tested.

The purpose of the current study was to expand these results by increasing the number of surfactant and extreme/ocular corrosive (GHS Category 1) chemicals tested with and without ascorbic acid to determine the accuracy of the detection of “nonirritants” (GHS Category NC), irritants (GHS Categories 2B and 2A), and extreme/ocular corrosives (GHS Category 1), bringing the total number of chemicals tested in triplicate for the new updated method to 147 (59 UN GHS Category 1, 37 UN GHS Category 2, and 51 UN GHS NC).

## 2. Methods

The OS macromolecular test was conducted as previously described (Choksi et al., 2020; Lebrun et al., 2021a, 2022b) and is briefly described below.

### 2.1. OS protocol

The OS test is packaged as a complete kit (Lebrun Labs LLC, Anaheim, CA), which includes the OS “Active Agent” (AA) macromolecular reagent formulation (see Lebrun et al., 2022a for a description). As described in prior publications (Lebrun et al., 2021b, 2022a, 2022b), samples were initially evaluated using a set of Physiochemical Handling Procedure (PCHP) pretests (see Lebrun et al., 2022a). Specific physiochemical properties (solubility, buffering capacity, and foaming) of the material to be tested are measured during the pretest step. Based on these physiochemical properties, there are specific changes in the protocol that improve sensitivity and accuracy (Lebrun et al., 2021b, 2022a, 2022b). For substances with significant pH buffering, the buffering power was further evaluated, and the protocol was adjusted to match; testing of insoluble materials does not use membrane discs and surfactants are diluted.

For the updated version of the test, L-ascorbic acid at in vivo levels (530 μM, Sigma-Aldrich, Milwaukee, WI, Catalog number A5960) was added directly to the AA formulation (see Lebrun et al., 2022a). The updated version of OS with ascorbic acid has an updated prediction model (Table 1).

Besides the updates for the updated version with ascorbic acid, the standard protocol was followed; five increasing doses of test chemicals were titrated onto an “ocular disc,” which controls the delivery of the chemical to be tested as it enters the reagent mixture. After incubation at 31 °C, the OD is measured with a spectrophotometer. The resulting OD and pH values are compared with quality controls and a standard curve to generate a score (see Choksi et al., 2020 for procedure details and flow chart).

Scores are assigned classifications based on the older (Choksi et al., 2020; Lebrun et al., 2022a) or new unified OS prediction model (Lebrun et al., 2022a). For the new prediction model (Table 1), a score of 15 or less predicts GHS NC, and an OS score >45 predicts a GHS Category 1. Scores that fall within these cutoffs are predicted as GHS Category 2A/ 2B (Lebrun et al., 2022a).

In some cases (when the dose-response curve is nonlinear and for some buffering capacity test outcomes), the OS method does not differentiate between UN GHS Categories 1 and 2 (UN, 2021). Test chemicals that are predicted as Category 2/1 should only be used for GHS NC versus the rest analyses and must therefore be subsequently tested by another method to establish a definitive UN GHS Category 2 or 1 Classification. In other cases, the OS method cannot provide results, termed “criteria not met”” (CNM). CNM occurs when the photometric range of the spectrophotometer has been exceeded or when there is an inverse dose-response curve below the irritant cut-off (Lebrun et al., 2022a, 2022b).

### 2.2. Test chemicals

All test chemicals had existing publicly available in vivo “Draize” rabbit eye data to which OS results are compared (no new animal testing was performed). Specific references for in vivo classifications are provided as part of the tables. Newly tested chemicals are shown in Table 2A (GHS NC), Table 2B (GHS Categories 2B and 2A) and Table 2C (GHS category 1). Chemicals included a broad range of physiochemical properties and chemical classes. The new testing for the retrospective chemicals are for the updated OS (OS values have been previously reported, Choksi et al., 2020). Surfactants were selected by the OS validation management team for the prior study (Choksi et al., 2020) but were not tested as part of that study because surfactants were previously excluded based on the OS PCHP foaming test (Choksi et al., 2020). As shown in Table 2C, Forty-eight additional GHS Category 1 chemicals were newly tested, bringing the total number of GHS Category 1 chemicals tested in triplicate to 59, thereby allowing for a more significant evaluation of the ability of the test to detect GHS Category 1.

### 2.3. Statistics

For statistical evaluation, data for the newly tested chemicals were combined with data for the previously tested chemicals. The additional testing increased the *n* from 78 (Lebrun et al., 2022a) to 147 (total *n* for this paper). OS results were compared with the in vivo results to determine the predictive capacity. In vivo GHS classifications for each chemical were obtained from historical databases (no new in vivo testing was done) of live animal “Draize” test eye irritation/damage data (Draize et al., 1944); specific references for each chemical are provided as part of the tables (Tables 3A – 3D).

### 2.4. GHS NC vs. the rest calculations

The accuracy was calculated as the number of correctly predicted in vivo positives (GHS Category 2 or 1) predicted by OS to be positive (Category 2 or 1) added to the number of in vivo negatives (NC) identified by OS to be negative (NC) divided by the total number of chemicals. The sensitivity was calculated as the total number of in vivo Category 2 or 1 chemicals that were correctly predicted by OS to be Category 2 or 1 divided by the total number of in vivo Category 2 or 1 chemicals. The specificity was calculated as the total number of in vivo NC chemicals that were correctly predicted by OS to be NC divided by the total number of in vivo NC chemicals. The FN rate was calculated as the total number of in vivo Category 2 or 1 chemicals that were predicted by OS to be NC divided by the total number of in vivo positives (Category 2 or 1). The FP rate was calculated as the total number of in vivo NC chemicals predicted by OS to be Category 2 or 1 divided by the total number of in vivo negatives (NC).

### 2.5. GHS Category 1 vs. the rest calculations

The accuracy was calculated as the number of in vivo Category 1 chemicals predicted to be Category 1 by OS plus the number of not Category 1 chemicals predicted to be not Category 1 by OS divided by the total number of chemicals. The sensitivity was calculated as the total number of in vivo Category 1 chemicals that were correctly predicted by OS to be Category 1 divided by the total number of in vivo Category 1 chemicals. The specificity was calculated as the total number of in vivo Category 2 or NC chemicals that were correctly predicted by OS to be Category 2 or NC divided by the total number of in vivo Category 2 or NC chemicals. The FN rate was calculated as the number of in vivo Category 1 chemicals that were predicted by OS as NC or Category 2 divided by the total number of in vivo positives (Category 1). The FP rate was calculated as the total number of in vivo NC or Category 2 chemicals that were predicted by OS to be Category 1 divided by the total number of in vivo negatives (NC).

## 3. Results

Triplicate results for the OS and updated OS “transferability” and “application domain” studies have previously been published (Choksi et al., 2020; Lebrun et al., 2022b), and the consensus prediction for these triplicate results are provided as Supplemental Data Table S1. In this context, “consensus result” means the majority prediction of the three repeats (either 2/3 or 3/3). Consensus results for the past OS retrospective study (Choksi et al., 2020) are compared in Table 3A with the newly tested individual triplicate results for the same chemicals tested with the updated OS. Tables 3B and C show the new triplicate results for both OS and updated OS for the “expanded corrosive set” (38 additional GHS Category 1 chemicals) and “surfactant set” (12 surfactant chemicals), and additional chemicals (3D), respectively. All of the compiled consensus results were then used for predictivity analysis. Table 4 shows the in vivo results in the column on the left and the OS and updated OS results in the row at the top. This table allows for a comprehensive comparison of OS (Table 4A) and updated OS (Table 4B).

As shown in Tables 4A and 4B, NC overpredictions decreased for the updated OS compared with OS. For OS, there were 18 FPs and 3 CNM. Of the 18 FPs, 11 were overpredicted as Category 2B/2A, 6 as Category 1, and 1 as Category 2/1. For updated OS, there were 10 FPs and 2 CNM. Of the 10 FPs, 6 were overpredicted as Category 2 B/A, 2 as Category 1, and 2 as Category 2/1. 1,3-Di-isopropylbenzene (99–62-7) was predicted as Category 1 with OS and as Category 2/1 with updated OS. The following NC predictions that were overpredicted by OS as Category 2 were correctly predicted by updated OS as TNs: 1,9-decadiene (1647-16-1), 2,2-dimethyl-3-pentanol (3970-62-5), triethylene glycol (112–27-6), 2-(2-ethoxyethoxy)ethanol (111–90-0), n,*n*-dimethylguanidine sulfate (598–65-2), Tween 80 (9005–65-6), Tween 20 (9005–64-5), and styrene (100–42-5) (corrected from an OS Category 1 to NC with updated OS). 2-Ethoxyethyl methacrylate (2370-63-0) and sodium lauryl sulfate (3%) (151–21-3) were predicted by OS as Category 1 and by updated OS as Category 2. Also note that 1,5-hexadiene (592–42-7) was excluded by OS due to the lack of consensus between the repeats and was predicted as an NC with updated OS.

For both OS and updated OS, there were no Category 2 B/A under-predictions (see Tables 4A and 4B). However, the GHS in vivo Category 2 B/A overpredictions differed between OS and updated OS. For OS, 14 chemicals were predicted as Category 2 B/A, 20 were overpredicted as Category 1, 2 were predicted as Category 2/1, and 1 was CNM. For updated OS, 11 chemicals were predicted as Category 2 B/A, 20 chemicals were overpredicted as Category 1, 3 were predicted as Category 2/1, and 3 were CNM. n,n-Diethyl-m-toluamide (134–62-3) and chlorhexidine digluconate solution (18472–51-0) were predicted as Category 2 with OS and as Category 1 with updated OS. Maneb (solid) (12427–38-2) was predicted as a Category 1 with OS and CNM with *OS; blanks exceeded the acceptance criteria of OD 0.850. Sodium benzoate (532–32-1) was predicted as Category 1 with OS and Category 2/1 with updated OS due to a negative dose-response curve, indicating assay inhibition. Sodium lauroyl sarcosinate (10%) (137–16-6) was predicted as a Category 2 with OS and CNM with updated OS; the resulting score for updated OS (CNM) was under the irritancy cut-off and had a negative dose-response curve.

As shown in Table 4, a total of 59 GHS Category 1 chemicals were tested. For the OS GHS Category 1 chemicals (Table 4A), none were underpredicted as GHS NC, 8 were underpredicted as Category 2 B/A, 37 were correctly predicted as Category 1, 7 were predicted as Category 2/1, and 7 were CNM. For updated OS GHS Category 1 chemicals (Table 4B), none were underpredicted as GHS NC, 4 were underpredicted as Category 2 B/A, 44 were predicted as Category 1, 7 were predicted as Category 2/1, and 4 were CNM. These differences included diethylaminopropionitrile (5351–04-2), imidazole (288–32-4), and sodium perborate tetrahydrate (10486–00-7), which were CNM with OS and predicted as Category 1 with *OS. 2,5-Dimethylhexanediol (110—03—2) and butanedioic acid, sulfo-, 1,4-bis(2-ethylhexyl) ester, sodium salt (577–11-7) were underpredicted as Category 2 with OS and predicted as Category 1 with updated OS. When the mass of solid was reduced from 10% to 2.5% for the pretest procedure (to reduce the chance that colloid suspensions interfere with spectrophotometric readings) two additional test articles were classified as completely insoluble; floats (CiF) PCHP subprotocol. Paraformaldehyde (30525–89-4) was predicted as a Category 2 with OS and a Category 1 with updated OS. 2-Hydroxy isobutyric acid ethyl ester (80–55-7) was predicted as a Category 2 with OS and a Category 1 with updated OS. (3-Aminopropyl) triethoxy silane (919–30-2) was predicted as a Category 1 with OS and a Category 2 with updated OS. Cetyltrimethyl ammonium bromide (10%) (57–09-0) was predicted as a Category 2 with OS and a Category 1 with updated OS.

Tables 5A and 5B, respectively, show the predictive capacity for OS and updated OS for the detection of GHS NC versus the rest. For NC versus the rest, both OS and updated OS have a very high sensitivity (100%) and low FN rate (0%). The FP rate is cut almost in half for updated OS compared to OS (compare Tables 4A and 4B), which results in an improved accuracy for updated OS compared with OS (92.8% vs. 86.8%). Balanced accuracy corrects for the ratios of positives and negatives. The balanced accuracy for OS is 81.3% and the balanced accuracy for updated OS is 89.8%. The superior balanced accuracy of updated OS is best attributed to the improvement (reduction) in the updated OS FP rate compared to OS, since both have a zero FN rate (100% sensitivity).

Tables 5C and 5D show the predictive capacity for OS and updated OS, respectively, for the detection of GHS Category 1 versus the rest. For Category 1 versus the rest, the sensitivity was improved for updated OS compared with OS (91.7% vs. 82.2%, respectively). Also, the specificity was better for updated OS compared with OS (71.8% vs. 67.9%, respectively). These improvements resulted in a better accuracy of 79.4% for updated OS compared with an accuracy of 73.0% for the older version of OS.

## 4. Discussion

In this study, we assessed the ability of the original and updated versions of the OS test to predict the ocular irritation potential of 59 ocular “corrosives” (GHS Category 1), 37 ocular “irritants” (GHS Category 2), and 51 not classified as ocular irritant chemicals (GHS NC). This set of chemicals included surfactants representative of all GHS levels of classification. Remarkably, for all of the OS and updated OS studies, there has not been a consensus GHS NC versus irritant FN. This finding is in contrast to other nonanimal ocular irritation test predictive capacities for GHS NC, including the BCOP Laser-Light Based Opacitometer, ICE, EpiOcular^™^ Eye Irritation Test (EIT), Ocular Irritection^®^ (OI), and STE eye irritation tests (Lebrun et al., 2021a). As shown in Table 6A, other OECD-accepted nonanimal EITs have sensitivities that range from 88% to 97%. While the OECD Test Guideline 437 lists the BCOP OP-KIT as having a 100% sensitivity, in more recent studies, the BCOP (OP-KIT) was found to have a sensitivity closer to 88.0%–93.3% (OECD, 2020a; Lebrun et al., 2021a).

A goal of this study was to determine the OS and updated OS predictive capacity for the detection of extreme/corrosive chemicals (GHS Category 1) and thereby expand the application domain to this important class of chemicals. Table 5 shows the accuracy of 73.0% (Table 5C) for the original OS test, and Table 5D shows the accuracy of 79.4% for the updated OS. Table 6B compares these values of other EITs with those of the OECD guidelines. Compared with these other tests, updated OS has the highest sensitivity and balanced accuracy.

As shown in Table 7, the 8.3% GHS category 1 underprediction rate for the updated OS was for GHS Category 2; No GHS category 1 chemicals were underpredicted as UN GHS NC. Also shown in Table 7, there is a high overprediction rate for UN GHS Category 2 to be overpredicted as UN GHS Category 1. Therefore, while “not causing eye damage (Not UN GHS Category 1)” predictions have high sensitivity and can be accepted without further testing, UN GHS Category 1, CNM and Category 2/1 predictions would be subsequently tested using other adequately validated in vitro test(s). The current “state of the art” is to use multiple tests in a tiered-testing approach using a “bottom-up” or “top-down” series (Scott et al., 2010; Alépée et al., 2019a, 2019b; OECD, 2019). We propose that OS is better suited for a “bottom-up” GHS Category 1 strategy, which may find utility in cases where only a single test to rule out the potential for eye damage (GHS Category 1) can be conducted following a “bottom-up strategy.” Since the “top-down strategy” employs multiple tests with low sensitivity but high specificity to sequentially identify Category 1 (Scott et al., 2010; Alépée et al., 2019a, 2019b; OECD, 2019), in some cases, the requirement to conduct multiple tests may be viewed as complicated and expensive. In addition, there are situations where a quick answer about whether a material causes eye irritation or damage would be useful. Since OS is shelf stable and ready to use, and results can be obtained in <24 h, it fits well for cases when a single, sensitive test with a fast turn-around time can be used to identify chemicals that do not “irritate” (GHS NC) or do not damage (not GHS Category 1) the eye.

In summary, the OS application domain was expanded to include surfactants and ocular corrosives (GHS Category 1), and these studies confirm and extend our previous findings, showing that the antioxidant, ascorbic acid, improves the accuracy of the OS test by reducing the FP rate. The OS test has a high sensitivity and accuracy for the identification of materials that should be classified as GHS NC and chemicals that do not damage the eye (not Category 1). Based on these outcomes, the best use of OS within a tiered testing strategy may be: 1) to identify chemicals that do not induce serious eye damage (not UN GHS Category 1), that is, chemicals not to be classified as UN GHS Category 1 without further testing; and 2) to identify chemicals predicted to be UN GHS NC, that is, predicted not to cause eye irritation/serious eye damage without further testing; however, chemicals predicted to be UN GHS Category 1 would require additional information and/or testing to establish a definitive UN GHS Category classification.

## Supplementary Material

1

## Figures and Tables

**Table 1:** Updated OptiSafe GHS prediction model.

Irritation Score	GHS Classification
≤15	NC
>15–45	Category 2 B/A
>45	Category 1

GHS = Globally Harmonized System of Classification and Labeling of Chemicals; NC = Not classified.

**Table 2A:** GHS NC chemicals.

Chemical Name	CASRN	Sup.	Cat No.	Purity (%)	Physical Properties	PCHP	Chemical Class
Dioctyl ether	629–82-3	SiAl	249,599	99.0	Colorless liquid^3^, VP 0.0 ± 0.07 kPa at 25 °C^2^	MAα	Ether^6^
Di-n-propyl disulfide	629–19-6	SiAl	149,225	98.0	Colorless to pale yellow liquid^1^, VP 0.07 kPa^1^	MAα	Disulfide^6^
sec-Butylbenzene	135–98-8	SiAl	B90408	99.0	Colorless liquid^1^, VP 0.2 kPa at 25 °C^1^	MAα	Hydrocarbon (cyclic)^5^
Isopropyl myristate	110–27-0	SiAl	172,472	98.0	Colorless to pale yellow liquid^1^, VP 0.01 Pa at 25 °C^1^	Maα	Alkane, branched with secondary carbon; Carboxylic acid ester; Isopropyl^7^
1,5-Dibromopentane	111–24-0	SiAl	128,007	97.0	Colorless to yellow liquid^3^, VP 0.01 ± 0.05 kPa at 25 °C^2^	MAα	Alkyl halide^7^
2,4,5,6-Tetraaminopyrimidine sulfate salt	5392-28-9	SiAl	T3807	97.0	Yellow powder^3^	H	Amine; Heterocycle; Inorganic salt^5^
Polyoxyethylene hydrogenated castor oil (60E.O.)	61,788–85-0	SiAl	07076	n.a.	White to yellow paste^3^, VP 0.0 ± 0.2 kPa at 25 °C^2^	SA	Acylal; Alcohol; Allyl, Ether^6^
Tween 80	9005–65-6	SiAl	P8074	n.a.	Amber viscous liquid^1^, VP 0.0 ± 0.7 kPa at 25 °C^2^; VIS 300–500 cP at 25 °C^1^	SA	Acetal; Alcohol; Alkane, branched with secondary carbon; Alkene moiety; Allyl; Carboxylic acid ester; Ether moiety; Monosaccharide derivatives; Oxolane; Saturated heterocyclic fragment ^7^
Sodium lauryl sulfate (3%)	151–21-3	SiAl	436,143	≥99.0	White powder^3^, VP 0.0002 kPa at 20 °C^4^	SA	Carboxylic acid (salt)^5^
Cetylpyridinium bromide (0.1%)	140–72-7	SiAl	52,340	≥ 97.0	Off-white powder^3^	SA	Hetercyclic; Onium compound^5^
Tween 20	9005–64-5	SiAl	P1379	n.a.	Yellow viscous liquid^3^, VP 0.0 ± 0.7 kPa at 25 °C^2^	SA	Ester; Polyether^5^
Triton X-100 (1%)	9002-93-1	SiAl	X100	n.a	Colorless liquid^3^, VP 0.0 ± 0.2 kPa at 25 °C^2^	SA	Ether^5^
Myristyl myristate	3234-85-3	SCBT	sc-295508A	n.a.	White crystals^3^	CiF	Carboxylic acid ester^5^

**Table 2B:** GHS category 2B/2A chemicals.

Chemical Name	CASRN	Sup.	Cat No.	Purity (%)	Physical Properties^1^	PCHP	Chemical Class
1-Tetradecanol	112–72-1	SiAl	185,388	97.0	White pellets^1^, VP 0.01 Pa at 25 °C^1^	CiF	Alcohol^7^
Sodium benzoate	532–32-1	SiAl	71,300	≥99.0	White powder^1^, VP 3.9 × 10^−10^ Pa at 25 °C^1^	MAα	Aryl; Carboxylic acid^6^
Naphthalene-1,5-diol	83–56-7	SiAl	D115606	97.0	Grey powder^3^, VP 0.0 ± 0.1 kPa at 25 °C^2^	Maα	Fused carbocyclic aromatic; Naphthalene; Phenol^6^
Chlorhexidine digluconate solution	18,472–51-0	SiAl	C9394	20.0	Colorless liquid^3^	MAα	Aromatic heterocyclic halide; Aryl halide; Dihydroxyl group; Guanidine^6^
Sodium lauroyl sarcosinate (10%)	137–16-6	SiAl	61,745	≥97.0	White powder^3^, VP^4^ 0.002 kPa at 20 °C^4^	SA	Aliphatic amine, tertiary; Amine, tertiary; Carboxylic acid; Organic amide and thioamide^7^
Sodium deoxycholate (10%)	302–95-4	SiAl	D6750	≥97.0	White powder^3^, VP 0.01 kPa at 20 °C^4^	SA	Alcohol; Carboxylic acid (salt)^5^
Triton X-100 (5%)	9002-93-1	SiAl	X100	n.a.	Colorless liquid^3^, VP 0.0 ± 0.2 kPa at 25 °C^2^	SA	Ether^5^
Ethanolamine	141–43-5	SiAl	411,000	≥ 99.5	Colorless viscous liquid^1^, VP 53.0 Pa at 20 °C^1^, VIS 19.0 cP at 25 °C^1^	H	Alcohol; Amine, primary; Aliphatic amine, primary^7^
Cetylpyridinium bromide (1%)	140–72-7	SiAl	52,340	≥ 97	Off-white powder^3^	SA	Hetercyclic; Onium compound^5^
Sodium lauryl glucose carboxylate (and) lauryl glucoside	383,178–66-3 (110615–47-9)	Ross Org.	n.a.	n.a.	Viscous liquid^3^	SA	n.a.

**Table 2C:** GHS Category 1 chemicals.

Chemical Name	CASRN	Sup.	Cat No.	Purity (%)	Physical Properties^1^	PCHP	Chemical Class
Tetraethylene glycol diacrylate	17,831–71–9	SiAl	398,802	≥ 87.0	Pale yellow liquid^3^, VP 0.0 ± 0.1 kPa at 25 °C^2^	HMA	Acrylate; Ether^6^
Sodium oxalate	62–76-0	SiAl	71,800	≥ 99.5	White powder^3^	CiS	Carboxylic acid (salt)^5^
Promethazine hydrochloride	58–33-3	SiAl	P4651	≥ 97.0	White powder^3^	Maα	Amine/Amidine; Heterocyclic; Organic sulfur compound^5^
Tetraoctylammonium bromide	14,866–33-2	SiAl	294,136	98.0	White powder^3^	CiF	Quaternary ammonium salts^7^
Glycolic acid	79–14-1	SiAl	798,053	99.0	White powder^1^, VP 2.7 Pa at 25 °C^1^	H	Alcohol; Carboxylic acid^7^
Acetic Acid (10%)	64–19-7	SiAl	695,092	≥99.7	Colorless liquid^1^, VP 1.5 kPa at 20 °C^1^, VIS	H	Acetoxy; Carboxylic acid^7^
Sodium hydroxide (10%)	1310-73-2	SiAl	S8045	≥ 98.0	Colorless liquid^1^, VP 0 kPa^1^, VIS 4.0 cP at 350 °C^1^	H	Alkali^5^
Pyridine	110–86-1	SiAl	270,407	≥ 99.9	Colorless to yellow liquid^1^, VP 2.0 kPa at 20 °C^1^	HMA	Heterocyclic^5^
Trichloroacetic acid (30%)	76–03-9	SiAl	T6399	≥99.0	Off-white crystalline solid^1^, VP 133 Pa at 51 °C^1^	H	Carboxylic acid^5^
2,2-Dimethylbutanoic acid	595–37-9	SiAl	D152609	96.0	Colorless to faint green liquid^1^, VP 0.02 kPa^1^	HMA	Carboxylic acid^5^
Diethylethanolamine	100–37-8	SiAl	471,321	≥ 99.5	2.8 kPa at 20 °C^1^, VIS 5 cP at 20 °C^1^	H	Alcohol; Aliphatic amine, tertiary; Amine, tertiary^7^
m-Phenylene diamine	108–45-2	SiAl	P23954	99.0	Colorless to white solid crystals^1^, VP 133 Pa at 99.8 °C^1^	HMA	Amine, primary; Aniline; Aryl; Phenylenediamine, meta-^7^
Sodium salicylate	54–21-7	SiAl	S3007	≥ 99.5	White crystals^3^, VP 0.01 kPa at 20 °C^4^	Maα	Aryl; Carboxylic acid; Phenol^7^
Benzoic acid	65–85-0	SiAl	242,381	≥ 99.5	White crystalline solid^1^, VP 0.1 Pa at 25 °C^1^, VIS 1.3 cP at 130 °C^1^	HMA	Aryl; Carboxylic acid^6^
Tetrahydrofuran	109–99-9	SiAl	401,757	≥ 99.9	Colorless liquid1, VP 19.3 kPa at 20 °C^1^, VIS 0.5 cP at 20 °C^1^	Maα	Ether moiety; Oxolane; Saturated heterocyclic fragment^7^
Methylpentynol	77–75-8	SiAl	137,561	98.0	Pale yellow liquid^1^, VP 0.7 kPa^1^	Maα	Alcohol; Alkane, branched with tertiary carbon; Alkyne moiety^7^
1-Naphthalene acetic acid Na salt	61–31-4	FiSc	N000725G	≥ 96.0	White powder^3^	Maα	Carboxylic acid (salt); Polycyclic compound^5^
Dibenzoyl-*L*-tartaric acid	2743-38-6	FiSc	AAA1618122	99.0	White powder3	H	Carboxylic acid; Ester5
bis-(3-Aminopropyl)-tetramethyldisiloxane	2469-55-8	FiSc	AAL1729522	94.0	Colorless liquid^3^, VP 0.0 ± 0.07 kPa at 25 °C^2^	H	Aliphatic amine, primary; Amine, primary; Disiloxane7
4,4′ -(4,5,6,7-Tetrabromo-3H-2,1-benzoxathiol-3-ylidene)bis[2,6-dibromophenol] S,S-dioxide	4430–25-5	SiAl	199,311	85.0	Blue powder^3^, VP 0.0 ± 0.3 kPa at 25 °C^2^	HMA	Aromatic heterocyclic halide; Aromatic perhalogen carbons; Aryl halide; Benzoxathiole S-oxide; Phenol; Sulfonate ester^6^
2-Methylbutyric acid	116–53-0	SiAl	193,070	98.0	Colorless liquid^1^, VP 0.07 kPa at 20 °C	H	Alkane, branched with tertiary carbon; Carboxylic acid^7^
Paraformaldehyde	30,525–89-4	SiAl	158,127	95.0	White powder^1^, VP 0.2 kPa at 20 °C^1^, VIS 0.1 cP at 25 °C^1^	HMA	Alcohol
Methoxyethyl acrylate	3121-61-7	SiAl	408,913	98.0	Colorless liquid^3^, VP 2.3 kPa at 61 °C^4^	HMA	Acrylate; Alkene moiety; Carboxylic acid ester; Ether moiety^7^
2-Hydroxy isobutyric acid	594–61-6	SiAl	323,594	99.0	White crystals^1^, VP: 1.3 Pa^1^	H	Alcohol; Alkane, branched with tertiary carbon; Carboxylic acid^7^
alpha-Ketoglutaric acid	328–50-7	SiAl	K1750	≥ 98.5	White to pale yellow crystals^3^, VP 0.01 kPa at 25 °C^4^	H	Carboxylic acid; Ketone; Oxocarboxylic acid^7^
beta-Resorcylic acid	89–86-1	SiAl	D109401	97.0	White powder^3^, VP 0.0 ± 0.1 kPa at 25 °C^2^	H	n.a.
Sodium hydrogen sulfate	7681-38-1	SiAl	307,823	n.a.	White crystals^3^	H	n.a.
1-Naphthaleneacetic acid (solid)	86–87-3	SiAl	N0640	≥ 95.0	White crystals^3^, VP 0.0 ± 0.1 kPa at 25 °C^2^	HMA	Carboxylic acid; Polycyclic compound^5^
Quinacrine	69–05-6	SiAl	Q3251	≥ 90.0	Bright yellow powder^3^	Maα	Heterocyclic^5^
Benzenesulphonylchloride	98–09-9	SiAl	108,138	99.0	Colorless to slightly yellow powder^1^, VP 0.009 kPa at 25 °C^1^	H	Aryl; Sulfonyl halide^7^
1,2,4-Triazole, sodium salt	41,253–21-8	SiAl	197,645	n.a.	White powder^3^	HMA	Aryl; Triazole^6^
4-(1,1,3,3-Tetramethylbutyl)phenol	140–66-9	SiAl	290,823	97.0	White powder^1^, VP 4.7 × 10^−3^ kPa at 74 °C	CiF	Alkane, branched with quaternary carbon; Alkyl (hetero)arenes; Alkyl-, alkenyl-, and alkynyl (hetero)arenes; Aryl; Phenol; tert-Butyl^7^
1-Chloroctan-8-ol	23,144–52-7	SiAl	415,693	98.0	Colorless liquid^1^, VP 0.0 ± 0.1 kPa at 25 °C^2^	Maα	Alcohol; Alkyl halide^7^
2-Benzyl-4-chlorophenol	120–32-1	SiAl	548,618	95.0	White to light tan or pink flakes^1^, VP 0.01 kPa at 68 °C^1^	CiS	Aryl; Aryl halide; Phenol; Precursors quinoid compounds^7^
1,2-Benzisothiazol-3(2H)-one	2634-33-5	SiAl	561,487	97.0	White powder^1^, VP 3.7 × 10^−7^ kPa at 25 °C^1^	HMA	Benzthiazolinone/Benzoisothiazolinone^6^
Chlorhexidine	55–56-1	SiAl	282,227	≥ 99.5	White powder^1^, VP 2.6 × 10^−15^ kPa at 25 °C^1^	HMA	Amine/Amidine^5^
2-Hydroxy isobutyric acid ethyl ester	80–55-7	SiAl	E31200	98.0	Colorless liquid^3^, VP 0.2 ± 0.8 kPa at 25 °C^2^	HMA	Alcohol; Alkane, branched with tertiary carbon; Carboxylic acid ester^7^
Hydroxyethyl acrylate	818–61-1	SiAl	292,818	96.0	Colorless liquid^1^, VP 7.0 Pa at 25 °C^1^, VIS 5.2 cP at 15 °C^1^	HMA	Acrylate; Alcohol; Alkene moiety; Carboxylic acid ester^7^
(3-Aminopropyl)triethoxy silane	919–30-2	SiAl	440,140	99.0	Colorless liquid^1^, VP 3.3 kPa at 121 °C^1^	HMA	Aliphatic amine, primary; Alkoxysilane; Amine, primary; Silane^7^
2-Nitro-4-thiocyanatoaniline	54,029–45-7	SiAl	550,647	95.0	Gold powder^3^, VP 0.0 ± 0.1 kPa at 25 °C^2^	Maα	n.a.
Benzalkonium chloride (5%)	63,449–41-2	SiAl	12,060	≥ 95.0	Colorless viscous liquid^3^	SA	Onium compound^6^
Benzalkonium chloride (1%)	63,449–41-2	SiAl	12,060	≥ 95.0	Colorless viscous liquid^3^	SA	Inorganic salt; Onium compound^6^
Cetyltrimethyl ammonium bromide (10%)	57–09-0	SiAl	H6269	≥ 99.0	White powder^3^	SA	Organic salt; Onium compound^5^
2-Methylresorcinol	608–25-3	SiAl	302,589	98	White crystalline powder^4^	Maα	Aryl; Phenol; Precursors quinoid compounds; Alkyl (hetero)arenes; Alkyl-, alkenyl-, and alkynyl (hetero)arenes^5^
4-tert-Butylcatechol	98–29-3	SiAl	19,671	≥99	White powder^4^	HMA	Aryl; Phenol; tert-Butyl; Alkyl (hetero)arenes; Alkyl-, alkenyl-, and alkynyl (hetero)arenes^5^
Cetylpyridinium bromide (6%)	140–72-7	SiAl	52,340	≥97	Off-white powder^3^	SA	Hetercyclic; Onium compound^5^

GHS = Globally Harmonized System of Classification and Labeling of Chemicals; NC = Not classified; CASRN = Chemical Abstracts Service Registry Number; Sup. = Supplier; SiAl = Sigma-Aldrich (3050 Spruce Street, St. Louis, MO 63103); FiSc = Fisher Scientific (4500 Turnberry Drive, Hanover Park, IL 60133–5491); Ross Org. = Ross Organics; Cat. No = Catalog Number from the listed supplier; Purity (%) = purity as stated by supplier; VP = Vapor pressure; VIS = Viscosity; PCHP = Physiochemical Handling Procedures; MAα = Membrane Assay alpha; HMA = Moderate pH buffering; H = Extreme pH buffering; SA = Surfactant; CiF = Completely insoluble, floats.

1 = PubChem (https://pubchem.ncbi.nlm.nih.gov/);

2 = ChemSpider (www.chemspider.com); data generated from computational software if data unavailable on PubChem;

3 = Personal Observation;

4 = Sigma-Aldrich Material Safety Data Sheet;

5 = ICCVAM, 2010;

6 = EURL ECVAM, 2014;

7 = OECD Toolbox Profiler V4.4.1.

**Table 3A:** Results for Retrospective Study.

OptiSafe and Updated OptiSafe Consensus Results for “Retrospective” Phase							
#	Chemical Name (CASRN)	In vivo *GHS^1^*	OptiSafe	Updated OptiSafe^2^			
			Cons^1^	R1	R2	R3	Cons
1	Dioctyl ether (629–82-3)	*NC*	NC	1.4 (NC)	0.7 (NC)	4.2 (NC)	NC
2	Di-n-propyl disulfide (629–19-6)	*NC*	NC	2.3 (NC)	1.2 (NC)	1.3 (NC)	NC
3	sec-Butylbenzene (135–98-8)	*NC*	NC	4.2 (NC)	9.5 (NC)	4.9 (NC)	NC
4	Isopropyl myristate (110–27-0)	*NC*	NC	0.7 (NC)	6.2 (NC)	1.6 (NC)	NC
5	1,5-Dibromopentane (111–24-0)	*NC*	NC	3.6 (NC)	3.1 (NC)	2.3 (NC)	NC
6	2,4,5,6-Tetraaminopyrimidine sulfate salt (5392-28-9)	*NC*	2/1	36.9 (2/1)	38.8 (2/1)	38.4 (2/1)	2/1
7	Ethanolamine (141–43-5)	*2B*	2/1	23.4 (2/1)	22.3 (2/1)	22.3 (2/1)	2/1
8	1-Tetradecanol (112–72-1)	*2A*	1	59.0 (1)	72.2 (1)	56.5 (1)	1
9	Sodium benzoate (532–32-1)	*2A*	1	23.1 (2/1)	26.0 (2/1)	29.1 (2/1)	2/1
10	Naphthalene-1,5-diol (83–56-7)	*2A*	1	94.3 (1)	94.3 (1)	102.5 (1)	1
11	Chlorhexidine digluconate solution (18472–51-0)	*2A*	2	45.8 (1)	46.2 (1)	45.0 (2)	1
12	Tetraethylene glycol diacrylate (17831–71–9)	*1*	1	73.5 (1)	61.3 (1)	50.3 (1)	1
13	Sodium oxalate (62–76-0)	*1*	1	55.4 (1)	56.7 (1)	64.4 (1)	1
14	Promethazine hydrochloride (58–33-3)	*1*	1	172.2 (1)	159.6 (1)	126.8 (1)	1
15	Tetraoctylammonium bromide (14866–33-2)	*1*	1	80.3 (1)	51.1 (1)	56.7 (1)	1
16	Glycolic acid (79–14-1)	*1*	1	51.7 (1)	49.7 (1)	50.7 (1)	1
17	Acetic acid (10%) (64–19-7)	*1*	2/1	18.8 (2/1)	18.2 (2/1)	19.1 (2/1)	2/1
18	Sodium hydroxide (10%) (1310-73-2)	*1*	1	51.9 (1)	52.1 (1)	52.9 (1)	1

**Table 3B:** Results for the Corrosives Study.

OptiSafe and Updated OptiSafe Consensus Results for Corrosives								
#	Chemical Name (CASRN)	In vivo *GHS^1^*	OptiSafe	Updated OptiSafe				
			R1	Cons^1^	R1	R2	R3	Cons
19	Pyridine (110–86-1)	*1*	673.8 (1)	1	600.6 (1)	751.1 (1)	1063.9 (1)	1
20	Trichloroacetic acid (30%) (76–03-9)	*1*	2,754,228.7 (1)	1	74.8 (1)	71.4 (1)	72.1 (1)	1
21	2,2-Dimethylbutanoic acid	(595–37-9)	*1*	761.1 (1)	1 658.5 (1)	1070.0 (1)	1510.6 (1)	1
22	Diethylethanolamine (100–37-8)	*1*	3020.0 (2/1)	2/1	20.6 (2/1)	20.7 (2/1)	20.1 (2/1)	2/1
23	m-Phenylene diamine (108–45-2)	*1*	CNM	CNM	CNM	CNM	CNM	CNM
24	Sodium salicylate (54–21-7)	*1*	87.9 (1)	1	98.3 (1)	104.1 (1)	96.9 (1)	1
25	Benzoic acid (65–85-0)	*1*	640.5 (1)	1	196.4 (1)	239.7 (1)	171.3 (1)	1
26	Tetrahydrofuran (109–99-9)	*1*	44.1 (2)	2	27.3 (2)	26.6 (2)	37.3 (2)	2
27	Methylpentynol (77–75-8)	*1*	105.9 (1)	1	70.1 (1)	86.1 (1)	72.3 (1)	1
28	1-Naphthalene acetic acid Na salt (61–31-4)	*1*	350.3 (1)	1	108.6 (1)	112.6 (1)	91.8 (1)	1
29	Dibenzoyl-*L*-tartaric acid (2743-38-6)	*1*	83,176.4 (2/1)	2/1	42.7 (2/1)	43.3 (2/1)	43.5 (2/1)	2/1
30	bis-(3-Aminopropyl)-tetramethyldisiloxane (2469–55-8)	*1*	3311.3 (2/1)	2/1	17.2 (2/1)	19.7 (2/1)	19.2 (2/1)	2/1
31	4,4′ -(4,5,6,7-Tetrabromo-3H-2,1-benzoxathiol-3-ylidene)bis[2,6-dibromophenol] S,S-dioxide (4430–25-5)	*1*	CNM	CNM	CNM	CNM	CNM	CNM
32	2-Methylbutyric acid (116–53-0)	*1*	3981.1 (2/1)	2/1	21.8 (2/1)	22.7 (2/ 1)	23.4 (2/ 1)	2/1
33	Paraformaldehyde (30525–89-4)	*1*	38.0 (2)	2	65.0 (1)	38.3 (2)	47.8 (1)	1
34	Methoxyethyl acrylate (3121–61-7)	*1*	66.3	(1)	1 93.0	(1) 54.0	(1) 56.8 (1)	1
35	2-Hydroxy isobutyric acid (594–61-6)	*1*	234,422.9 (1)	1	54.4 (1)	54.8 (1)	53.3 (1)	1
36	alpha-Ketoglutaric acid (328–50-7)	*1*	1,905,460.7 (1)	1	71.6 (1)	72.8 (1)	70.7 (1)	1
37	beta-Resorcylic acid (89–86-1)	*1*	51,286.1 (2/1)	2/1	41.1 (2/ 1)	39.7 (2/1)	39.1 (2/1)	2/1
38	Sodium hydrogen sulfate (7681-38-1)	*1*	5,370,318.0 (1)	1	84.6 (1)	85.7 (1)	84.1 (1)	1
39	1-Naphthaleneacetic acid (solid) (86–87-3)	*1*	141.4 (1)	1	121.7 (1)	203.1 (1)	141.3 (1)	1
40	Quinacrine (69–05-6)	*1*	264.3 (1)	1	159.8 (1)	349.6 (1)	122.7 (1)	1
41	Benzenesulphonylchloride (98–09-9)	*1*	1096.5 (2/1)	2/1	CNM	18.8 (2/1)	20.5 (2/1)	2/1
42	1,2,4-Triazole, sodium salt (41253–21-8)	*1*	206.6 (1)	1	46.0 (1)	40.2 (2)	46.1 (1)	1
43	4-(1,1,3,3-Tetramethylbutyl)phenol (140–66-9)	*1*	110.8 (1)	1	158.0 (1)	139.3 (1)	127.6 (1)	1
44	1-Chloroctan-8-ol (23144–52-7)	*1*	47.4 (2)	2	30.8 (2)	32.7 (2)	32.0 (2)	2
45	2-Benzyl-4-chlorophenol (120–32–1)	*1*	79.8 (1)	1	155.5 (1)	147.6 (1)	142.0 (1)	1
46	1,2-Benzisothiazol-3(2H)-one (2634-33-5)	*1*	200.5 (1)	1	171.0 (1)	311.7 (1)	271.3 (1)	1
47	Chlorhexidine (55–56-1)	*1*	121.0 (1)	1	271.8 (1)	331.8 (1)	350.0 (1)	1
48	2-Hydroxy isobutyric acid ethyl ester (80–55-7)	*1*	54.9 (2)	2	120.6 (1)	157.6 (1)	120.7 (1)	1
49	Hydroxyethyl acrylate (818–61-1)	*1*	76.3 (1)	1	105.3 (1)	79.3 (1)	57.8 (1)	1
50	(3-Aminopropyl)triethoxy silane (919–30-2)	*1*	143.8 (1)	1	44.4 (2)	33.6 (2)	31.5 (2)	2
51	2-Nitro-4-thiocyanatoaniline (54029–45-7)	*1*	503.6 (1)	1	CNM	243.3 (1)	182.8 (1)	1

**Table 3C:** Results for Surfactants.

OptiSafe and Updated OptiSafe Consensus Results for Corrosives										
#	Chemical Name (CASRN)	In vivo *GHS^1^*	OptiSafe				Updated OptiSafe			
			R1	R2	R3	Cons^1^	R1	R2	R3	Cons
52	Polyoxyethylene hydrogenated castoroil (60E.O.) (61788–85-0)	NC	9.4 (NC)	10.9 (NC)	8.0 (NC)	NC	5.3 (NC)	5.0 (NC)	4.7 (NC)	NC
53	Tween 80 (9005–65-6)	NC	13.9 (2)	13.6 (2)	12.2 (NC)	2	6.3 (NC)	6.5 (NC)	6.3 (NC)	NC
54	Sodium lauryl sulfate (3%) (151–21-3)	NC	52.7 (1)	50.1 (1)	47.9 (1)	1	35.9 (2)	32.0 (2)	37.4 (2)	2
55	Cetyl pyridinium bromide (0.1%) (140–72-7)	NC	6.8 (NC)	6.2 (NC)	6.1 (NC)	NC	3.8 (NC)	3.9 (NC)	3.9 (NC)	NC
56	Tween 20 (9005–64-5)	NC	29.0 (2)	27.1 (2)	14.4 (2)	2	8.3 (NC)	8.9 (NC)	9.2 (NC)	NC
57	Triton X-100 (1%) (9002-93-1)	NC	8.1 (NC)	6.7 (NC)	8.6 (NC)	NC	4.3 (NC)	4.8 (NC)	4.5 (NC)	NC
58	Sodium lauroyl sarcosinate (10%) (137–16-6)	*2A*	14.1 (2)	26.1 (2)	13.8 (2)	2	CNM	CNM	CNM	CNM
59	Sodium deoxycholate (10%) (302–95-4)	*2A*	64.6 (1)	61.1 (1)	61.0 (1)	1	50.9 (1)	51.1 (1)	49.6 (1)	1
60	Triton X-100 (5%) (9002-93-1)	*2A*	63.9 (1)	55.1 (1)	78.8 (1)	1	46.4 (1)	50.8 (1)	44.6 (2)	1
61	Benzalkonium chloride (5%) (63449–41-2)	*1*	66.0 (1)	64.5 (1)	62.1 (1)	1	58.5 (1)	60.8 (1)	61.4 (1)	1
62	Benzalkonium chloride (1%) (63449–41-2)	*1*	26.9 (2)	26.3 (2)	26.6 (2)	2	29.6 (2)	30.1 (2)	30.7 (2)	2
63	Cetyltrimethyl ammonium bromide (10%) (57–09-0)	*1*	44.4 (2)	47.6 (2)	44.9 (2)	2	42.4 (1)	46.2 (1)	53.4 (1)	1

**Table 3D:** Results for additional chemicals.

OptiSafe and Updated OptiSafe Consensus Results for Additional Chemicals								
#	Chemical Name (CASRN)	In vivo *GHS^1^*	OptiSafe		Updated OptiSafe			
			R1	Cons	R1	R2	R3	Cons
64	Myristyl myristate (3234-85-3)	*NC*	11.0 (NC)	NC	12.8 (NC)	2.9 (NC)	14.4 (NC)	NC
65	Sodium lauryl glucose carboxylate (and) lauryl glucoside	*2A*	64.7 (1)	1	596.8 (1)	599.4 (1)	611.6 (1)	1
66	Cetylpyridinium bromide (1%) (140–42-7)	*2A*	27.4 (2)	2	27.0 (2)	26.4 (2)	28.0 (2)	2
67	Cetylpyridinium bromide (6%) (140–42-7)	*1*	53.4 (1)	1	55.3 (1)	54.8 (1)	51.2 (1)	1
68	4-Tert-butylcatechol (98–29-3)	*1*	CNM	CNM	CNM	CNM	CNM	CNM
69	2-methylresorcinol (608–25-3)	*1*	CNM	CNM	CNM	CNM	CNM	CNM

CASRN = Chemical Abstracts Service Registry Number; GHS = Globally Harmonized System of Classification and Labeling of Chemicals; In vivo GHS = GHS classifications based on the retrospective Draize rabbit data (reference classification); R = Repeat; Cons = Consensus; NC and (NC) = GHS Not classified; CNM = Criteria not met; 2 and (2) = GHS Category 2B and 2A (reversible irritation); 1 and (1) = GHS Category 1 (extreme/corrosive).

**Table 4A:** OptiSafe GHS classifications.

Category		OptiSafe					
		1	2 B/A	NC	2/1	CNM	Total
in vivo *GHS*	1	37	8	0	7	7	59
	2 B/A	20	14	0	2	1	37
	NC	6	11	30	1	3	51
	Total	63	33	30	10	11	147

**Table 4B:** Updated OptiSafe GHS classifications.

Category		Updated OptiSafe					
		1	2 B/A	NC	2/1	CNM	Total
in vivo *GHS*	1	44	4	0	7	4	59
	2 B/A	20	11	0	3	3	37
	NC	2	6	39	2	2	51
	Total	66	21	39	12	9	147

In vivo and OptiSafe (4A) and Updated OptiSafe (4B) predicted GHS classifications. GHS = Globally Harmonized System of Classification and Labeling of Chemicals; CNM = Criteria not met; NC = No category.

**Table 5 A.** OptiSafe GHS NC vs. Rest. B. Updated OptiSafe GHS NC vs. Rest. C. OptiSafe GHS Cat. 1 vs. Rest. D. Updated OptiSafe GHS Cat. 1 vs. Rest.

GHS NC vs. Rest			
A. OptiSafe		B. Updated OptiSafe	
Statistic	OptiSafe	Statistic	Updated OptiSafe
Sensitivity	100.0% (88/88)	Sensitivity	100.0% (89/89)
Specificity	62.5% (30/48)	Specificity	79.6% (39/49)
FN Rate	0.0% (0/88)	FN Rate	0.0% (0/89)
FP Rate	37.5% (18/48)	FP Rate	20.4% (10/49)
Accuracy	86.8% (118/136)	Accuracy	92.8% (128/138)
Bal. Accuracy	81.3%	Bal. Accuracy	89.8%
GHS Cat.1 vs Rest			
C. OptiSafe		D. Updated OptiSafe	
Statistic	OptiSafe	Statistic	Updated OptiSafe

Sensitivity	82.2% (37/45)	Sensitivity	91.7% (44/48)
Specificity	67.9% (55/81)	Specificity	71.8% (56/78)
FN Rate	17.8% (8/45)	FN Rate	8.3% (4/48)
FP Rate	32.1% (26/81)	FP Rate 28.2% (22/78)	
Accuracy	73.0% (92/126)	Accuracy	79.4% (100/126)
Bal. Accuracy	75.1%	Bal. Accuracy	81.8%

Cat. = Category; FN Rate = False-negative rate; FP Rate = False-positive rate; Bal. Acc. = Balanced Accuracy; NC = Not classified. Table 4 explains the differences in n. GHS NC versus the rest used 2/1 predictions and GHS NC versus the rest does not use 2/1 predictions. See text for details.

**Table 6A:** Predictivity of OptiSafe and Updated OptiSafe Compared to Eye Irritation Tests with OECD Guidelines for Detection of NC versus the Rest.

Test Method	Sensitivity	Specificity	Accuracy	Bal. Accuracy
STE^1^	88%	81%	85%	84.5%
BCOP (LLBO)^2^	94%	55%	83%	74.5%
EpiOcular^3^	96%	63%	80%	79.5%
ICE^4^	97%	76%	88%	86.5%
Ocular Irritection^5^	91%	59%	75%	75.0%
OptiSafe	100.0%	62.5%	86.8%	81.3%
Updated OptiSafe	100.0%	79.6%	92.8%	89.8%

**Table 6B:** Predictivity of OptiSafe and Updated OptiSafe Methods Compared to Eye Irritation Tests with OECD Guidelines for Detection of GHS Category 1 versus the Rest.

Test Method	Sensitivity	Specificity	Accuracy	Bal. Accuracy
STE^1^	49%	99%	83%	74.0%
BCOP (LLBO)^2^	76%	79%	78%	77.5%
ICE^4^	53%	93%	83%	73.0%
Ocular Irritection^5^	54%	81%	75%	67.5%
OptiSafe	82.2%	67.9%	73.0%	75.1%
Updated OptiSafe	91.7%	71.8%	79.4%	81.8%

OECD = Organization for Economic Cooperation and Development; BCOP = Bovine Corneal Opacity and Permeability; LLBO = Laser-light based opacitometer; ICE = Isolated Chicken Eye; STE = Short Time Exposure; FNR = False-negative rate; FPR = False-positive rate.

1 = OECD, 2020b;

2 = OECD, 2020a;

4 = OECD, 2018;

5 = OECD, 2019b.

**Table 7:** Consensus Performance of the Updated OptiSafe Test using the 3 × 3 Matrix Showing Correct, Under- and Overpredictions per UN GHS Category.

UN GHS Categories	Updated OptiSafe – Predicted Categories (n/N%)		
	Cat 1 (n)	Cat 2 (n)	No Cat (n)
Cat 1 (*N* = 48)	91.7% (44)	8.3% (4)	0.0% (0)
Cat 2 (*N* = 31)	64.5% (20)	35.5% (11)	0.0% (0)
No Cat (*N* = 47)	4.3% (2)	12.8% (6)	83.0% (39)

UN = United Nations; GHS = Globally Harmonized System of Classification and Labeling of Chemicals; Cat = Category; N = total number of GHS in vivo classified chemicals; n = total number of OptiSafe predicted GHS classified chemicals.

## Data Availability

Data will be made available on request.

## Abbreviations:

AA: Active Agent
CASRN: Chemical Abstracts Service Registry Number
CNM: Criteria Not Met
EIT: Eye Irritation Test
FN: False Negative
FP: False Positive
GHS: Globally Harmonized System of Classification and Labeling of chemicals
ICCVAM: Interagency Center for the Evaluation of Alternative Toxicological Methods
NC: No Category
NTP: National Toxicology Program
OD: Optical Density
OECD: Organization for Economic Cooperation and Development
OS: OptiSafe
*OS: Updated OptiSafe
PCHP: Physiochemical Handling Procedure
ROS: Reactive Oxygen Species
UN: United Nations.
