# Supplementary material for: Expansion of the application domain of a macromolecular ocular irritation test (OptiSafe™)
Source: Toxicol In Vitro. Author manuscript; Available in PMC 2023 Feb 1. (PMC9802687; doi:10.1016/j.tiv.2022.105515)
Supplement: 1 [file NIHMS1851472-supplement-1.docx]

**Supplemental Tables**

Table S1A. Consensus Results for Past Transferability Study

| **OptiSafe and Updated OptiSafe Consensus Results for Past "Transferability" Phase** | | | | |
| --- | --- | --- | --- | --- |
| **#** | **Chemical Name (CASRN)** | ***In vivo GHS^1^*** | **OptiSafe** | **Updated OptiSafe** |
|  |  |  | **Cons^1, 2^** | |
| 1 | 1,3-Di-iso-propylbenzene (99-62-7) | *NC* | 1 | 2/1 |
| 2 | n-Hexyl bromide (111-25-1) | *NC* | NC | NC |
| 3 | iso-Octyl acrylate (29590-42-9) | *NC* | NC | NC |
| 4 | Glycerol (56-81-5) | *NC* | NC | NC |
| 5 | 1,9-Decadiene (1647-16-1) | *NC* | 2 | NC |
| 6 | Di-iso-butyl ketone (108-83-8) | *NC* | NC | NC |
| 7 | 1-Bromo-4-chlorobutane (6940-78-9) | *NC* | NC | NC |
| 8 | 1,6-Dibromohexane (629-03-8) | *NC* | NC | NC |
| 9 | n-Octyl bromide (111-83-1) | *NC* | NC | NC |
| 10 | Propylene glycol (57-55-6) | *NC* | NC | NC |
| 11 | 2,4-Pentanediol (625-69-4) | *NC* | NC | NC |
| 12 | Potassium tetrafluoroborate (14075-53-7) | *NC* | NC | NC |
| 13 | 4,4-Methylene bis-(2,6-ditert-butyl)phenol (118-82-1) | *NC* | CNM | CNM |
| 14 | 2,2-Dimethyl-3-pentanol (3970-62-5) | *NC* | 2 | NC |
| 15 | 2-Methyl-1-pentanol (105-30-6) | *2B* | 2 | 2 |
| 16 | Sodium chloroacetate (3926-62-3) | *2B* | 1 | 1 |
| 17 | Isobutyraldehyde (78-84-2) | *2B* | 1 | 1 |
| 18 | Camphene (79-92-5) | *2B* | 2 | 2 |
| 19 | Ammonium nitrate (6484-52-2) | *2A* | 2 | 2 |
| 20 | 3,3-Dithiodipropionic acid (1119-62-6) | *2B* | 1 | 1 |
| 21 | Isobutanol (78-83-1) | *2A* | 1 | 1 |
| 22 | Dibenzyl phosphate (1623-08-1) | *2A* | 1 | 1 |
| 23 | Propasol solvent P (1569-01-3) | *2A* | 2 | 2 |
| 24 | Methyl cyanoacetate (105-34-0) | *2A* | 1 | 1 |
| 25 | n-Butanol (71-36-3) | *1/2A* | 1 | 1 |
| 26 | 3,4-Dichlorophenyl isocyanate (102-36-3) | *1* | 1 | 1 |
| 27 | p-Tert-butylphenol (98-54-4) | *1* | 1 | 1 |

Table S1B. Consensus Results for Past Application Domain Study

| **OS and *OS Consensus Results for Past "Application Domain" Phase** | | | | |
| --- | --- | --- | --- | --- |
| **#** | **Chemical Name (CASRN)** | ***In vivo GHS^1^*** | **OptiSafe** | ***OptiSafe** |
|  |  |  | **Cons^1, 2^** | |
| 28 | Cyclopentasiloxane (541-02-6) | *NC* | NC | NC |
| 29 | Ethylene glycol diethyl ether (629-14-1) | *NC* | 2 | 2 |
| 30 | Hexane (110-54-3) | *NC* | NC | NC |
| 31 | 2-Ethylhexylthioglycolate (7659-86-1) | *NC* | NC | NC |
| 32 | iso-Propyl bromide (75-26-3) | *NC* | NC | NC |
| 33 | 1,2,6-Hexanetriol (106-69-4) | *NC* | NC | NC |
| 34 | 3-Methoxy-1,2-propanediol (623-39-2) | *NC* | NC | NC |
| 35 | Triethylene glycol (112-27-6) | *NC* | 2 | NC |
| 36 | *Triphenyl phosphite (101-02-0) | *NC* | 1 | 1 |
| 37 | 2-Ethoxyethyl methacrylate (2370-63-0) | *NC* | 1 | 2 |
| 38 | Hexamethyldisiloxane (107-46-0) | *NC* | NC | NC |
| 39 | Hexyl cinnamic aldehyde (101-86-0) | *NC* | NC | NC |
| 40 | p-Methyl thiobenzaldehyde (3446-89-7) | *NC* | 1 | 1 |
| 41 | Triclocarban (101-20-2) | *NC* | CNM | CNM |
| 42 | Ethyl acetate (141-78-6) | *NC* | 2 | 2 |
| 43 | 2,4-Pentanedione (123-54-6) | *NC* | 2 | 2 |
| 44 | Dodecane (112-40-3) | *NC* | NC | NC |
| 45 | 2-(2-Ethoxyethoxy)ethanol (111-90-0) | *NC* | 2 | NC |
| 46 | n,n-Dimethylguanidine sulfate (598-65-2) | *NC* | 2 | NC |
| 47 | 1,4-Dibromobutane (110-52-1) | *NC* | NC | NC |
| 48 | 3-Phenoxybenzyl alcohol (13826-35-2) | *NC* | 2 | 2 |
| 49 | Styrene (100-42-5) | *NC* | 1 | NC |
| 50 | 1,5-Hexadiene (592-42-7) | *NC* | CNM | NC |
| 51 | Xylene (1330-20-7) | *NC* | NC | NC |
| 52 | n,n-Diethyl-m-toluamide (134-62-3) | *2B* | 2 | 1 |
| 53 | 3-Chloropropionitrile (542-76-7) | *2B* | 1 | 1 |
| 54 | Isopropyl acetoacetate (542-08-5) | *2B* | 1 | 1 |
| 55 | *n*-Butanal (123-72-8) | *2B* | 1 | 1 |
| 56 | Ethyl-2-methyl acetoacetate (609-14-3) | *2B* | 1 | 1 |
| 57 | Maneb (solid) (12427-38-2) | *2B* | 1 | CNM |
| 58 | 6-Methyl purine (2004-03-7) | *2B* | 2/1 | 2/1 |
| 59 | Isopropanol (67-63-0) | *2A* | 2 | 2 |
| 60 | 2-Amino-3-pyridinol (16867-03-1) | *2A* | CNM | CNM |
| 61 | Allyl alcohol (107-18-6) | *2A* | 1 | 1 |
| 62 | Cyclopentanol (96-41-3) | *2A* | 1 | 1 |
| 63 | n-Hexanol (111-27-3) | *2A* | 2 | 2 |
| 64 | gamma-Butyrolactone (96-48-0) | *2A* | 2 | 2 |
| 65 | n-Octanol (111-87-5) | *2A* | 2 | 2 |
| 66 | Methyl acetate (79-20-9) | *2A* | 2 | 2 |
| 67 | 2,6-Dichlorobenzoyl chloride (4659-45-4) | *2A* | 1 | 1 |
| 68 | Acetone (67-64-1) | *2A* | 2 | 2 |
| 69 | Methylthioglycolate (2365-48-2) | *1* | 1 | 1 |
| 70 | Diethylaminopropionitrile (5351-04-2) | *1* | CNM | 1 |
| 71 | Imidazole (288-32-4) | *1* | CNM | 1 |
| 72 | Sodium perborate tetrahydrate (10486-00-7) | *1* | CNM | 1 |
| 73 | 2,5-Dimethylhexanediol (110-03-2) | *1* | 2 | 1 |
| 74 | Butanedioic acid, sulfo-, 1,4-bis(2-ethylhexyl) ester, sodium salt (577-11-7) | *1* | 2 | 1 |
| 75 | Cyclohexanol (108-93-0) | *1* | 1 | 1 |
| 76 | Lactic acid (50-21-5) | *1* | 1 | 1 |
| 77 | Protectol PP (80-54-6) | *1* | 1 | 1 |
| 78 | Lauric acid (143-07-7) | *1* | 1 | 1 |

Table S1. Consensus predictions for the OptiSafe (OS) and Updated OptiSafe (*OS) test methods. Consensus predictions were obtained from triplicate repeats for Updated OptiSafe. Updated OptiSafe predictions determined based on prediction model (Table 1). For OptiSafe, consensus predictions are from coded triplicate repeats each from three labs from the transferability study. OptiSafe predictions based on the prediction model reported in Choksi et al., 2020. Full results previously published (1 = Choksi et al., 2020; 2 = Lebrun et al., 2022). CNM = Criteria Not Met; CASRN = Chemical Abstracts Service Registry Number; GHS = Globally Harmonized System of Classification and Labeling of Chemicals; In vivo GHS = GHS classifications based on the retrospective Draize rabbit data (reference classification); NC = GHS Not classified; 2 = GHS Category 2B combined with 2A; 1= GHS Category 1 = Ocular corrosive.
